# Using long-term ranging patterns to assess within-group and between-group competition in wild mountain gorillas

**DOI:** 10.1186/s12898-020-00306-6

**Published:** 2020-07-16

**Authors:** Nicole Seiler, Martha M. Robbins

**Affiliations:** Department of Primatology, Max Planck Institute for Evolutionary Anthropology, Deutscher Platz 6, 04103 Leipzig, Germany

**Keywords:** *Gorilla beringei beringei*, Mountain gorillas, Intraspecific competition, Home range size, Home range fidelity, Home range exclusivity, Competitive advantage, Intergroup aggression, Mate defense

## Abstract

**Background:**

Competition within and between social groups determines access to resources and can be inferred from space use parameters that reflect depletion of food resources and competitive abilities of groups. Using location data from 1998 to 2017, we investigated within- and between-group competition in 12 groups of wild mountain gorillas (*Gorilla beringei beringei*). As within-group feeding competition is expected to increase with group size, an increase in group size is predicted to lead to an increase in the size of annual home ranges and core areas, but to a decrease in fidelity (reuse of an area). Due to asymmetries in competitive abilities, larger groups are expected to have higher exclusivity (degree of non-shared space) of annual home ranges and core areas than smaller groups.

**Results:**

We found evidence of within-group feeding competition based on a positive relationship between group size and both annual home range and core area size as well as a negative relationship between group size and core area fidelity. Additionally, fidelity of core areas was lower than of home ranges. Between-group competition was inferred from a trend for groups with more members and more males to have more exclusive home ranges and core areas. Lastly, annual core areas were largely mutually exclusive.

**Conclusions:**

Our study suggests that non-territorial, group-living animals can have highly dynamic, long-term avoidance-based spacing patterns, both temporally and spatially, to maintain annual core area exclusivity among groups while concurrently shifting these areas annually within overlapping home ranges to avoid resource depletion. Despite ranging in larger home ranges and core areas, larger groups were able to maintain more exclusive ranges than smaller groups, suggesting a competitive advantage for larger groups in between-group competition in a non-territorial species. Together, these findings contribute to understanding how social animals make behavioral adjustments to mitigate the effects of intraspecific competition.

## Background

Access to resources, such as food and mates, influences individual fitness [1]. Because resources are limited in space and time, group-living individuals face competition for access to resources with their group members as well as with neighboring groups [2, 3]. Feeding competition can take the form of scramble competition, where resources are exploited by the individual or group that arrives at a resource first, or as contest competition, in which one individual or group has a competitive advantage over another [3–5]. Competition for food resources leads to differences in the energetic status among members within a group or between groups [6]. Scramble competition for food within groups results in reduced foraging efficiency for all group members [2, 4] and hence is considered a cost of group living [5]. Between-group scramble competition can lead to energetic costs for all members in both groups [2, 7], whereas between-group contest competition results in only one group suffering energetic costs due to loss of access of resources [2, 7–9]. In contrast, mating competition is more intense among males than females in mammals and it can increase a male’s short-term and long-term reproductive success [6]. Competition for food and mates can be reflected in the size and utilization patterns of a group’s home range (area used to survive and reproduce [10]) and core area (area of intense utilization within a home range, which contains the biologically most relevant resources [11, 12]). Although within-group and between-group competition are expected to act concurrently, very few studies have investigated both at the same time [13].

According to the ecological constraints model, within-group feeding competition is expected to increase as group size increases, which necessitates groups to increase home range size and daily travel distance to meet the higher energetic requirements of additional group members. Hence, individuals in larger groups have to expend more energy than individuals in smaller groups [14, 15]. Positive relationships between group size and both home range and core area size are the primary markers used to infer scramble competition within groups in the wild [16–18], although such scramble competition may be mitigated by dispersal of males and/or females. In particular, female dispersal allows groups to adjust their size based on food availability and feeding competition, with females being able to move to smaller groups and experience less feeding competition [19–21]. In female-philopatric species, however, group size cannot decrease easily when feeding competition increases, possibly leading to stronger relationships between groups size and home range size.

The relationship between group size and home range size may be quadratic instead of linear because groups need to balance the trade-off between high within-group competition faced by large groups and high between-group competition faced by small groups, resulting in intermediate-sized groups having an optimal space use strategy in the form of smaller home ranges and/or shorter daily travel distances than small or large groups [22, 23]. Overall, the relationship between group size and home range size is sometimes disputed, especially in folivorous species that rely on abundant food resources as their groups are assumed to not be limited by the availability of food resources (e.g. [7, 18, 24]), and the non-linear relation between home range size and group size has rarely been tested.

Intraspecific competition for food and mates can also be measured with other behavioral indicators. Specifically, fidelity and exclusivity of home ranges and core areas reflect depletion of food resources and competitive abilities of groups but these variables have only seldom been examined [25–28]. Home range fidelity, which is the tendency of animals to return to and reuse previously used areas [29], could improve fitness of groups via increased foraging efficiency by allowing them to evaluate the quality, distribution and predictability of habitats over space and time [29–31]. However, it may be more efficient energetically to shift a range because depletion of food resources within a home range may lower foraging efficiency [32], especially as group size increases [33, 34]. Although also dependent on regeneration rates of food plants, variation in home range fidelity across time could be a valuable behavioral indicator of within-group scramble competition [27].

For non-territorial species, the level of between-group competition can be reflected in the degree of shared space among neighbors. In contrast to territorial species, non-territorial species that have overlapping home ranges are not expected to benefit from exclusive access to food resources resulting from ownership advantages or from intergroup dominance in between-group contest competition [8, 35]. Intergroup dominance, which usually arises from asymmetries in competitive abilities due to an advantage of having more members or more males [36–38], can increase the quality of the dominant group’s home range or the area of exclusive access [8, 39, 40]. This may be due to direct food defense by males to attract females (resource defense polygyny [39, 41, 42]) or as a consequence of direct mate defense (“hired guns” [42, 43]). Although non-territorial species are not expected to benefit from ownership advantages, they may still benefit from better access to resources in part of their home range [26, 44, 45]. However, for non-territorial species, it remains largely unclear whether groups have a competitive advantage in intergroup competition and how this is expressed.

To infer within- and between-group competition for food and mates in a non-territorial social mammal, we investigated the impact of group size on three parameters of space use in wild Bwindi mountain gorillas: size, fidelity and exclusivity of annual home ranges and core areas. The 2 populations of mountain gorillas are found in Bwindi Impenetrable National Park, Uganda, and in the Virunga Volcanoes of Uganda, Rwanda, and the Democratic Republic of Congo. Mountain gorillas live in cohesive social units consisting of one or more adult males, several adult females and their offspring (mean group size = 11) [46, 47]. Gorilla females disperse, which gives them the possibility to respond to an increase in within-group feeding competition by moving into smaller groups where there should be less competition [21]. The 2 populations feed predominantly on herbaceous vegetation, which is available throughout their range and throughout the year [48, 49]. Overall food biomass is higher in the Virungas than in Bwindi, which seems to be reflected in differential competitive regimes in the 2 populations [50]. Little evidence of costs of within-group feeding competition is apparent in the Virunga gorilla groups [51, 52] and group size has no clear effect on female reproductive success [53]. In contrast, Bwindi gorillas appear to experience some within-group feeding competition as groups reduce the frequency of revisits to particular areas as group size increases, although neither monthly home range size nor daily travel distance is affected by group size [54]. Within Bwindi, herbaceous food plants, the gorillas’ main diet, show spatial variation in availability despite little seasonal variability [55]. Assuming similar biomass regeneration rates for Bwindi as in the Virunga Volcanoes, food renewal rates, i.e. the time needed for the regeneration of biomass, are between 240 and 270 days [56]. Therefore, long-term studies, assessing spacing patterns on an annual scale, may best reflect the impact of depletion and regeneration of food resources (see [57]).

Competition between gorilla groups is mainly due to mating competition [58], with males being the main participants and females transferring to neighboring groups during intergroup encounters [59, 60]. Despite a high degree of home range overlap among neighboring groups in both populations [26, 52], intergroup encounters occur infrequently [54] and a recent 1 year study in Bwindi showed that groups have largely mutually exclusive core areas [26]. These core areas, in which the gorillas spend 50% of their time, contain higher herbaceous food availability than the rest of the home ranges. Such a spacing pattern appears to result from active avoidance among neighboring groups and seems to be stimulated by strong between-group competition for mates [26].

Using long-term ranging patterns of 12 groups over the course of 19 years (median = 6, range 1–19 years), we investigate how Bwindi gorillas concurrently adjust their behavior to compensate for the costs of both within- and between-group competition in the long term, complementing a 1 year study that investigated these competitive patterns in the short term [26, 54]. We tested the following predictions (Table 1):

**Table 1:** Overview of the predictions and results for within- and between-group competition in Bwindi mountain gorillas

Testing competitive regime	Predictor variable	Response variable	Prediction	Results
Within-group	Group size	Home range size	Positive effect	Positive effect
Within-group	Group size squared	Home range size	U-shape	ns
Within-group	Group size	Core area size	Positive effect	Positive effect
Within-group	Group size squared	Core area size	U-shape	ns
Within-group	Group size	Home range fidelity	Negative effect	ns
Within-group	Group size	Core area fidelity	Negative effect	Negative effect
Between-group	Core area (yes/no)	Home range and core area fidelity	Higher core area than home range fidelity	Higher home range than core area fidelity
Between-group	Core area (yes/no)	Home range and core area exclusivity	Higher core area than home range exclusivity	Higher core area than home range exclusivity
Between-group	Group size	Home range exclusivity	Positive effect	Trend for positive effect
Between-group	Number of males	Home range exclusivity	Positive effect	ns
Between-group	Group size	Core area exclusivity	Positive effect	Trend for positive effect
Between-group	Number of males	Core area exclusivity	Positive effect	Trend for positive effect

All response variables are annual

*ns* not significant

In accordance with the ecological constraints model [14, 15], we predicted a positive relationship between group size and both annual home range and core area size. Alternatively, we predicted a non-linear relationship (U-shaped) between both annual home range and core area size and group size because intermediate-sized groups may have an optimal space use strategy [22]. As there is greater resource depletion as group size increases [33, 34], we predicted a negative relationship between group size and both annual home range and core area fidelity between consecutive years. We decided to investigate fidelity of home ranges and core areas between consecutive years because there is low temporal variation in food availability and food renewal rates are expected to be between 240 and 270 days (assuming similar food renewal rates for Bwindi as in the Virunga Volcanoes [56]). Because highly used core areas of Bwindi gorillas were shown to be largely mutually exclusive and to contain higher herbaceous food availability than the rest of the home ranges in a 1 year study [26], we predicted that annual core area fidelity will be higher than annual home range fidelity across time.

Due to asymmetries in competitive abilities [8, 39] and because males are the active participants in intergroup encounters [59, 60], we expected that larger groups and groups with more males will maintain more exclusive (i.e. less overlap among neighbors) annual home ranges and core areas than smaller groups and groups with fewer males. Lastly, in a 1 year study Bwindi gorilla groups appeared to actively avoid competition with neighbors, resulting in shared home ranges but exclusive core areas [26], so we predicted that annual core areas will be more exclusive than annual home ranges across time.

## Results

### Home range and core area size

**Fig. 1:**
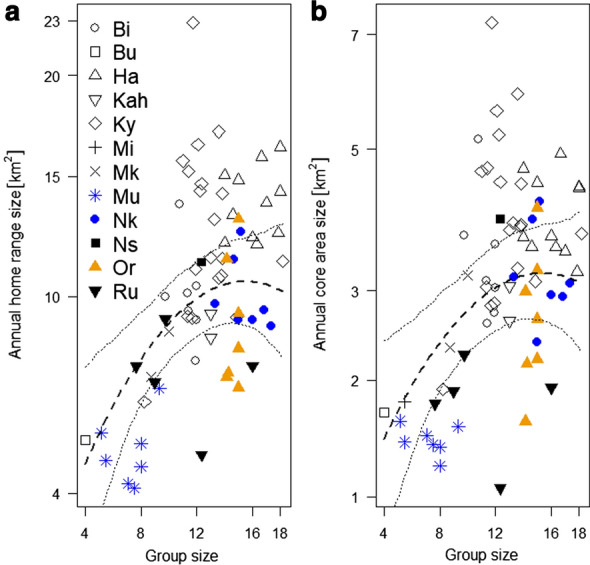
Impact of group size on **a** Annual home range size (90% kernel home range) and **b** Annual core area size (50% kernel home range) in Bwindi mountain gorillas. The response variables were square-root-transformed. The dashed and dotted lines indicate the fitted influence of the predictor on the response and its confidence interval, respectively

Bwindi mountain gorillas had a mean annual home range size of 10 km^2^ ± SE 0.44 (range 4.1–22.9 km^2^; see Additional file 1). When testing for the effect of group size and a quadratic value of group size (squared group size) on annual home range size, we found the full-null model comparison to show a trend (likelihood ratio test: χ^2^ = 4.945, df = 2, *p* = 0.084). The effect of group size was a trend (Est ± SE = 0.158 ± 0.069, *p* = 0.092) and the quadratic term was not significant (Est ± SE = − 0.090 ± 0.042, *p* = 0.108). The statistical model indicated that increasing the group size from 12 to 13 weaned individuals would increase the annual home range size by 2.5% (dashed line in Fig. 1a). According to the R^2^ value from a post-hoc linear regression, group size (and its quadratic term) accounted for 36.3% of the variance in the annual home range size.

The Bwindi mountain gorillas had a mean annual core area size (50% contours of the fixed kernel density estimation) of 3 km^2^ ± 0.15 (range 1.1–7.2 km^2^; see Additional file 1). When testing for the effect of group size and a quadratic value of group size on annual core area size, we found a significant difference between the full model and the null model (χ^2^ = 6.444, df = 2, *p* = 0.040). The effect of group size was a trend (Est ± SE = 0.084 ± 0.042, p = 0.065) and so was the quadratic term (Est ± SE = − 0.051 ± 0.026, *p* = 0.067). Visual inspection indicated that both the annual home range size and the mean annual core area size were predicted by the models to increase monotonically throughout most of the observed range of group sizes, before leveling off and then declining at the largest group sizes (Fig. 1). Results weakened in 2 additional models that included location as a category variable for the spatial variability in food availability (Additional file 2).

### Home range and core area fidelity

**Fig. 2:**
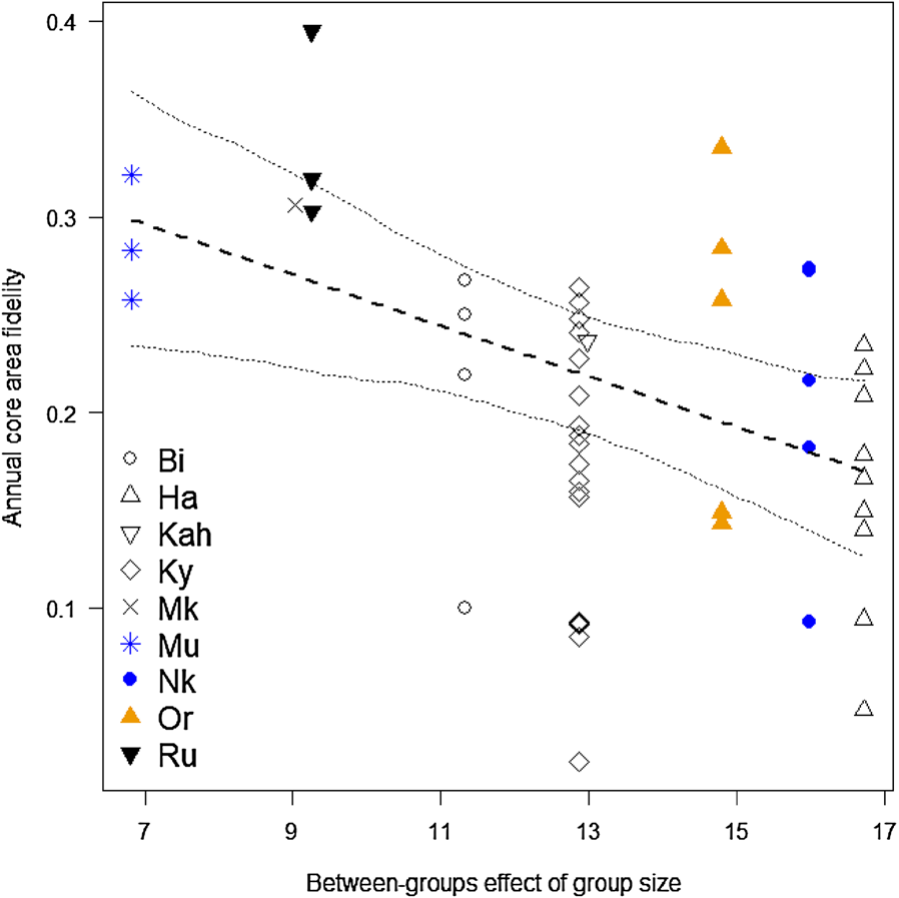
Impact of the between-groups effect of group size (= the mean of annual group size per group to account for the high variation in group size both among groups and within groups across time [89]) on annual core area fidelity (Bhattacharyya affinity of consecutive 50% annual kernel home ranges) in Bwindi mountain gorillas. The dashed and dotted lines indicate the fitted influence of the predictor on the response and its confidence interval, respectively

**Fig. 3:**
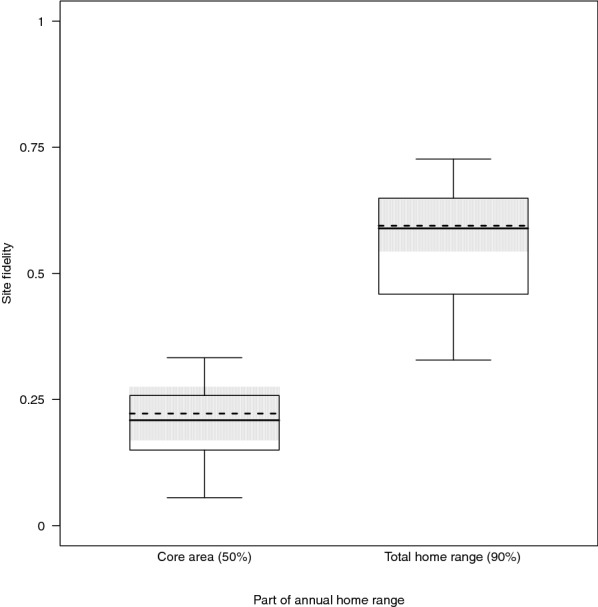
Annual home range and core area fidelity in Bwindi mountain gorillas. Influence of core area (yes/no) on the site fidelity (Bhattacharyya affinity). The dashed line indicates the fitted influence of the predictor on the response. The grey shaded areas depict bootstrapped 95% confidence intervals of the model

Examining the effect of group size on annual home range and core area fidelity (Bhattacharyya affinity = BA), we found that the mean BA for the annual home ranges was 0.6 ± 0.02 (range 0.31–0.79) and 0.2 ± 0.01 (range 0.02–0.39) for the annual core areas. There was no significant effect of group size on the BA for the annual home ranges (χ^2^ = 2.397, df = 2, *p* = 0.302), but it had a significant effect on the annual core area BA (χ^2^ = 6.137, df = 2, *p* = 0.047). Variation in group size among groups was as high as the variation within groups across time. We found a negative effect of the between-groups effect on the BA for the annual core area (Est ± SE = − 0.036 ± 0.013, χ^2^ = 5.873, *p* = 0.015; Fig. 2), whereas the within-groups effect had no apparent impact (Est ± SE = − 0.005 ± 0.010, χ^2^ = 0.253, *p* = 0.615). Against our prediction, we found that annual home range fidelity was significantly higher than annual core area fidelity (Est ± SE = − 0.372 ± 0.019, χ^2^ = 35.288, *p* < 0.001; Fig. 3).

### Home range and core area exclusivity

**Fig. 4:**
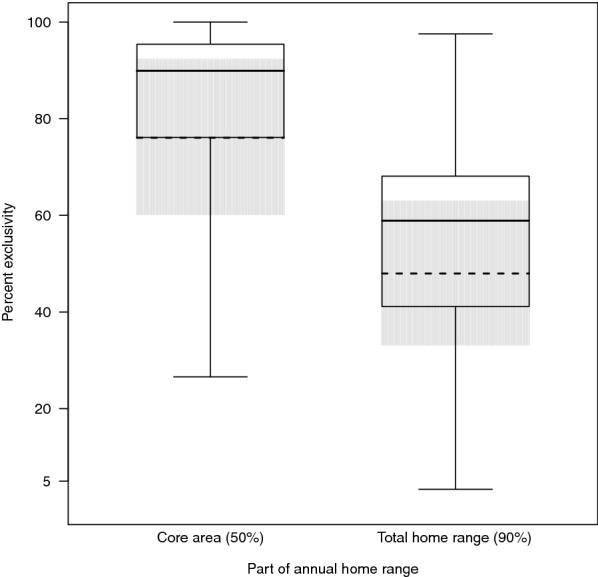
Percent exclusively used part of the annual home ranges and core areas in Bwindi mountain gorillas. Influence of core area (yes/no) on the percent of exclusivity. The dashed line indicates the fitted influence of the predictor on the response. The grey shaded areas depict bootstrapped 95% confidence intervals of the model

Bwindi gorillas used on average 54% ± 4.44 (range 3.1–98.9%) of their home ranges exclusively and on average 83% ± 3.58 (range 20.7–100%) of their core areas exclusively. The size of the exclusively used part of the annual home range tended to increase as group size increased (Est ± SE = 4.210 ± 1.841, χ^2^ = 3.342, df = 1, *p* = 0.068). For example, the statistical model indicated that increasing the group size from 12 to 13 weaned individuals would increase the size of the exclusively used part of the annual home range by 11.9%. According to the R^2^ value from a post-hoc univariate linear regression, group size accounted for 56.8% of the variance in the size of the exclusively used part of the annual home range. The number of males in a group did not have a significant effect on the size of the exclusively used part of its annual home range (Est ± SE = 1.445 ± 1.323; χ^2^ = 0.784, df = 1, *p* = 0.376). For the annual core area, we found that the size of the exclusively used part tended to increase as group size increased (Est ± SE = 1.823 ± 0.937, χ^2^ = 3.034, df = 1, *p* = 0.082) and as the number of males increased (Est ± SE = 1.928 ± 0.800, χ^2^ = 3.199, df = 1, *p* = 0.074). Lastly, annual core areas were more exclusive than annual home ranges (Est ± SE = 28.083 ± 5.120, χ^2^ = 14.963, *p* < 0.001; Fig. 4).

## Discussion

We found evidence of within- and between-group competition in Bwindi mountain gorillas using a long-term data set and variables rarely examined (fidelity and exclusivity of annual home ranges and core areas). The positive relationship between group size and both annual home range and core area size and the negative relationship between group size and core area fidelity suggest that increasing group size results in increased within-group feeding competition. We found some evidence that Bwindi gorillas also experienced between-group competition, as indicated by a trend for groups with more members and/or more males to have more exclusive home ranges and core areas. This suggests a competitive advantage for groups with more members and males, a relationship typically found in territorial species ([8, 9, 61] but see [44, 45]). Additionally, annual core areas were largely mutually exclusive across time (i.e. had considerably less overlap than annual home ranges), suggesting that groups avoid competition with neighboring groups in the long term, which might ultimately be due to mating competition.

### Within-group feeding competition

In accordance with the ecological constraints model [14, 15], both home range and core area size increased as group size increased on an annual scale. We found that small gorilla groups suffered costs from between-group competition by having less exclusive home ranges and core areas as well as that larger groups experienced costs from within-group competition. However, the combined linear effects of both within- and between-group competition did not result in the expected U-shaped relationship between group size and annual home range or core area size [19, 20]. Instead, we found evidence of a concave relationship, with the annual home range and the core area size leveling off and then declining at the larger group sizes. Similar patterns have been reported for the travel times and distances of the Virunga gorillas, which were attributed to the costs of within-group feeding competition for the smaller groups and the benefits of between-group feeding competition for the largest group [62]. Concave patterns were not observed, however, in the home ranges of those Virunga gorilla groups [52].

In Bwindi gorillas, neither daily travel distance nor monthly home range size increased significantly as group size increased [54] (see Table 2). Variation in the spatial availability of herbaceous vegetation might be small, such that on the scales of daily and monthly movement patterns groups may have found alternative strategies to mitigate the effect of within-group scramble competition, such as increasing group spread [16, 17, 63]. However, on the larger scale of annual movement, larger groups adjusted to greater within-group feeding competition and thus experienced higher energetic costs compared to smaller groups. Including location of groups as a proxy for food availability did not result in an increase in effect size, suggesting that it was not an accurate reflection of food availability on the scale that the gorillas experience. Bwindi gorillas only use a small proportion of their annual home range on a monthly basis (10 versus 1 km^2^; Additional file 1, [54]) and not increasing these monthly range sizes as group size increases should lead to higher depletion of food resources within these areas. To avoid these depleted areas, gorillas may shift their monthly home ranges within the annual home range, with larger groups having presumably less overlap among monthly home ranges than smaller groups, leading to an increase of the annual home ranges and core areas as group size increases.

**Table 2:** Overview of results investigating within- and between-group competition in Bwindi gorillas inferred from space use parameters across several spatial and temporal scales

Predictor variable	Group size	Local gorilla population density^e^	Intergroup encounter (yes/no)	Previous use by neighboring gorilla groups	Number of males	Core area (yes/no)
Response variable						
Daily travel distance^a^	No	No	Yes	–	–	
Monthly home range size^a^	No	Yes	No	–	–	
Revisit frequency to each part of the annual home range^a, c^	Yes	Yes	No	–	–	
Annual home range size	Yes	–	–	–		
Annual home range fidelity	Yes	–	–	–	–	
Annual home range exclusivity	Yes	–	–	–	Yes	Yes
Utilization of areas^b,d^	–	–	–	Yes	–	

^a^Taken from Seiler et al. [54]

^b^Taken from Seiler et al. [26]

^c^Corresponds to the number of times that each group of gorillas (n = 13) entered each 500 × 500 m grid cell within a group’s home range during a one year study period

^d^Corresponds to the distance travelled by each gorilla group (n = 10) in each 500 × 500 m grid cell during a one year study period

^e^This variable represents the weighted size of the Bwindi mountain gorilla population except the group. Therefore, the larger this estimate, the more neighboring gorillas can be found near a group

Group size also affected inter-annual core area fidelity, providing further evidence of within-group scramble competition. As group size increased core area fidelity decreased, presumably due to depletion of resources in relation to regeneration rates [27], but there was no effect on home range fidelity. Additionally, core area fidelity was lower than home range fidelity, again presumably due to depletion of food resources in the much smaller but highly utilized core areas. High home range fidelity but low core area fidelity suggests that groups shifted their annual core areas within stable annual home ranges. If groups deplete the resources available in their home range, they may need to move elsewhere. But if they are surrounded by other groups that also deplete their own home ranges, there is no guarantee that they should find more food if they move. In addition, they would pay the cost of having to search for their food and memorize the food distribution in new areas. Thus, there are reasons why large groups, which deplete their resources faster than small groups, may not move their annual home range more often.

Assuming similar biomass regeneration rates for Bwindi as in the Virunga Volcanoes (240–270 days [56]), these 2 rotational systems (shifting both monthly ranges and annual core areas within annual home ranges) may allow for regeneration of food resources in those areas. This also might ensure high food availability in the largely mutually exclusive core areas in the long term, which would be in concordance with a previous study that found higher food availability of core areas compared to home ranges in the short term [26]. Furthermore, such a forage rotation system follows predictions of the optimal foraging theory, which predicts animals should forage in areas that offer the highest average rate of energy intake [64, 65].

### Between-group mating competition

For non-territorial species that have overlapping home ranges, the level of between-group competition can be reflected in the degree of shared space among neighbors. Our results lend support to our predictions for between-group competition, as indicated by a trend for greater exclusivity of home ranges and core areas as group size and the number of males increases. Having more exclusive access to a home range and core area should lead to more exclusive and higher access to food resources (see [38, 62]). This should result in a difference in energetic status among groups and suggests that larger groups have a competitive advantage in intergroup competition, a pattern typically found in territorial species ([36, 66] but see [44, 45]). This reduced cost of between-group feeding competition for larger groups [2] could buffer the costs of increased within-group feeding competition [5, 67].

Bwindi mountain gorilla groups maintain core area exclusivity in the long term as annual core areas were more mutually exclusive than the respective home ranges. Groups used on average 83% of their core areas exclusively, whereas only 54% of their home ranges were exclusive. Together, this provides further support to the results of a 1 year study, which found exclusive use of core areas by non-territorial Bwindi mountain gorilla groups, suggesting that neighbors actively avoid each other [26].

Three proximate mechanisms that may allow groups to maintain this exclusivity by avoiding each other in the short term, may also work in the long term: remembering the locations of intergroup encounters to avoid these areas, using chest beats as a long-distance signal to locate neighbors and using signs of foraging to avoid previously used areas [26] (see also [66]). One ultimate mechanism for the observed avoidance-based spacing pattern might be male mate defense. In mountain gorillas, males have been considered as “hired guns”, in which males defend their mates and offspring by keeping extragroup males away, thereby indirectly defending food resources within these areas for the male’s group members [26, 43, 68]. This is in contrast to males exhibiting resource defense polygyny as found in several primate species, in which males indirectly defend females via resource defense [39, 42, 69], although both strategies can lead to high-quality home ranges.

We found a trend for groups with more males to have more exclusive core areas than groups with fewer males, which suggests a competitive advantage of additional males for directly defending mates and offspring within a group as well as indirectly defending food resources (see also [38]). Bwindi females residing in multimale groups might ultimately increase their reproductive success as an increasing number of males can provide increasingly exclusive core areas and thus exclusive access to food resources [2, 45, 67] (but see [70]).

## Conclusion

Through an investigation of long-term ranging patterns that reflect competition within and between groups, we found that despite having overlapping home ranges, mountain gorilla groups have largely mutually exclusive core areas that they shift on an annual scale to minimize the effect of within-group feeding competition. Furthermore, our results show that as group size increases, home range and core area size increases as well. Increasingly large home ranges should become more difficult to defend [71, 72] and overlap with neighbors is expected to increase [73]. Consequently, increasing home range size should lead to an increasing loss of food resources to neighbors [73]. Unexpectedly, we found that larger groups that range in larger home ranges and core areas had less overlap with their neighbors and hence were able to monopolize food resources in the exclusively used parts, thereby reducing the amount of food lost to neighbors. Together, this suggests that gorillas have evolved a highly dynamic avoidance-based spacing pattern, both temporally and spatially.

Through an investigation of the effect of group size on several space use parameters in a non-territorial species, our results show that while an increase in group size is beneficial for between-group competition, it is costly in terms of within-group feeding competition. Although many studies investigate within- or between-group competition independently (e.g. [9, 17]), we found evidence that both competitive regimes act simultaneously and animals adapt their behavioral response to balance the costs and benefits of between- and within-group competition concurrently [13] (see Table 2). These results stress the value of investigating both competitive regimes simultaneously across several scales to gain a comprehensive understanding of the various behavioral adjustments social animals exhibit to compensate for the costs of intraspecific competition.

## Methods

### Study site and data collection

**Fig. 5:**
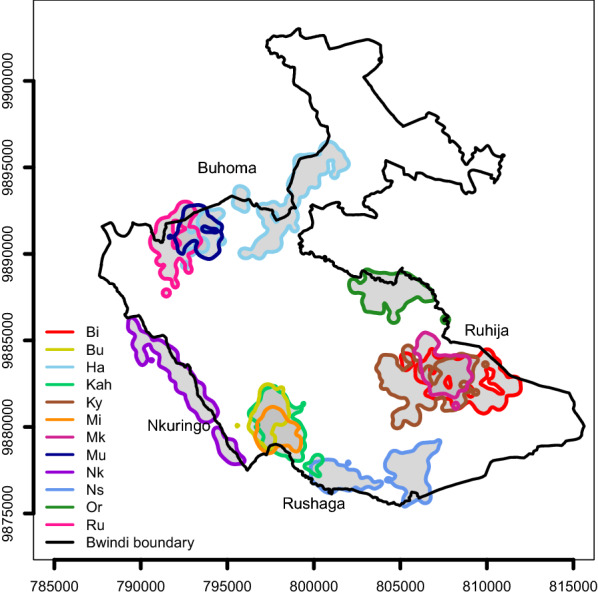
Location of the mountain gorilla study groups’ kernel home ranges (90% contours) in Bwindi Impenetrable National Park, Uganda (0.88–1.13ºN; 29.58–29.83ºE), and the four general locations where the groups range (Buhoma, Ruhija, Rushaga and Nkuringo). Because annual home ranges are quite stable in their location over time, we only used location data for the groups from 2012 except for group Mk, who formed after a fission in 2016, and for which data were taken from 2016, to show the location of the home ranges. The axes show UTM coordinates (in zone 35 M) so the distances between tick marks represent 5000 m. The map was generated by the authors

**Fig. 6:**
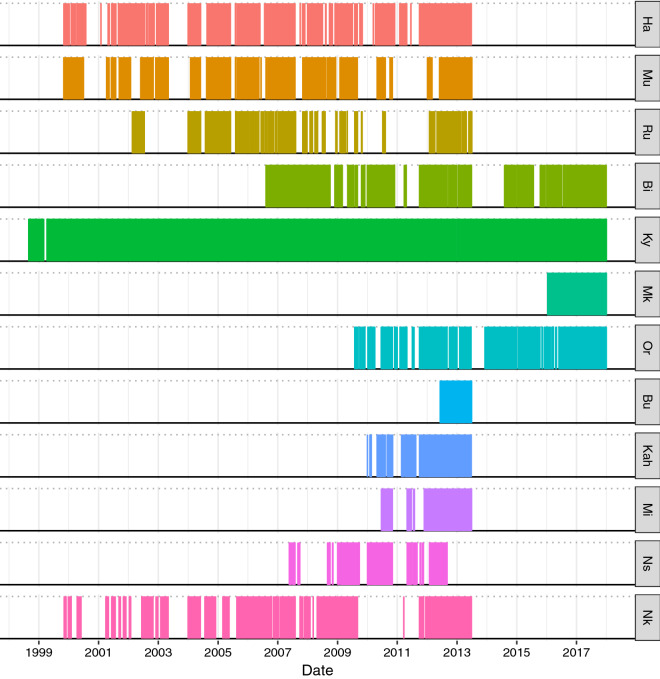
Location data coverage of Bwindi mountain gorillas per group and year. Each observation day is represented by a thin vertical line (n_days_ = 21,099). Groups Ha, Mu and Ru ranged around Buhoma; groups Bi, Ky, Mk and Or around Ruhija; groups Bu, Kah, Mi and Ns around Rushaga and group Nk around Nkuringo. Group Mk fissioned from group Ky in February 2016 and group Bu fissioned from group Kah in August 2012

We used location and demographic data on 12 groups of habituated mountain gorillas in Bwindi Impenetrable National Park, Uganda, from 1998 to 2017 (Fig. 5). Most of the location data from the groups habituated for tourism were taken from the long-term records of the Uganda Wildlife Authority. Permission for use of the data was obtained from the division of research and monitoring department of Uganda Wildlife Authority and access to the data can be obtained from them. All groups were monitored daily by park staff and in part by researchers to ascertain group membership. Mean annual group size was defined as the average number of weaned individuals per group and year (mean = 13, range 4.0–18.2 individuals), whereas the mean number of males (> 12 years of age; e.g. [74]) was defined as the average number of adult males per group and year (mean = 3, range 1.0–5.8 males). Using handheld global positioning system (GPS) units (GARMIN), one to several location points were recorded for each observation day and group. The number of observation days per year differed among groups, so location data coverage varied among groups and years (Fig. 6). Therefore, we randomly selected one location point per day (see also [26]).

### Response variables

#### Home range and core area size

Using the fixed kernel density estimation [75] and one location point per day, we determined annual home ranges (90% contours) and core areas (50% contours) per group applying the adehabitatHR package [76] in R 3.4.3 [77]. The kernel density estimation produces utilization distributions, which are probability distributions that describe the groups’ use of space [78]. As the number of observation days varied per group and year, we determined the minimum number of observation days needed for the annual home range size to reach an asymptote per group and year. This differed largely among years and groups, so we used only years with at least 125 observation days per group and randomly selected 125 observation days per group and year (n = 70) to estimate annual home range and core area sizes. We fixed the bandwidth to *h* = 200 because it creates annual home range estimates with relatively little fragmentation [26, 52], which is expected for Bwindi gorillas moving on average 975 m a day [54].

#### Home range and core area fidelity

The Bhattacharyya affinity (BA) [79] compares the overall similarity between utilization distributions [80, 81]. Using the annual kernel home ranges and core areas and the package adehabitatHR [76] in R 3.4.3 [77], we determined the BA between 2 consecutive years for each group (n = 49). Values range between zero and one, with one indicating identical utilization distributions and zero indicating no similarity between utilization distributions.

#### Home range and core area exclusivity

For each group and year, we determined both the size and the percentage of the annual home range and core area that was not shared with any neighboring group. To calculate the exclusively used part of the annual kernel home range and core area per group, we subtracted the shared parts from the annual kernel home ranges and core areas (n = 32; for more details see [26]). We included only years for which we had data on all habituated groups that ranged in the same area. We could not include the unhabituated gorilla groups in our analysis because the locations of their home ranges can only be approximated using few data points from the Bwindi censuses (e.g. [47, 82]). However, previous analyses have shown that it is unlikely that the habituated and unhabituated groups share notable amounts of the same ranges and hence we assume that excluding the unhabituated groups did not have a large effect on our results [26]. For data processing and analyses, we used the packages spatstat [83], splancs [84] and SDMTools [85] in R 3.4.3 [77].

### Models and statistical analyses

#### Models

We used linear mixed models (LMMs) [86] to quantify the effects of group size and group size squared (quadratic) on both annual home range and core area size (square-root-transformed; for both n = 70 observations from 12 groups and as much as 19 years). We included the general location the study groups ranged in as a control predictor as a factor with four levels (Buhoma, Ruhija, Nkuringo and Rushaga; Fig. 5) in 2 additional models as a proxy to control for the spatial variation in food availability among the different locations [55]. By doing so, we accounted for the possibility that larger groups range in areas with higher food availability than smaller groups [87]. We also investigated if the inclusion of the Nkuringo Group, which was observed primarily in uncultivated land outside the park and was observed to feed on palatable crops on 25% of observation days [88], had a notable effect on the results in comparison to other groups. It did not, so we retained it in all analyses (see Additional file 3).

We fitted 2 LMMs [86] to examine the effect of group size on annual home range and core area fidelity (BA estimates; for both n = 49 observations from 9 groups and as much as 18 years). For the test predictor group size, we determined the mean of the annual group sizes for the 2 corresponding consecutive years because variation between years was very small. As the variation in group size among groups was as high as the variation within groups across time, the mean of group size per group (= between-groups variation) and group size centered to a mean of zero per group (= within-groups variation) were included as test predictors in the model [89]. To test whether core area fidelity was higher than home range fidelity, we used a LMM [86] (n = 98 observations from 9 groups, as much as 18 years and 49 combinations of group and year). The response variable was the BA estimates [79] of both the annual home ranges and core areas, a measure that compares the overall similarity between utilization distributions. As a test predictor, we included whether the estimate originated from the core area or not as a factor with 2 levels (yes = core area and no = home range).

To investigate whether both group size and the number of males influenced the size of the exclusively used part of both the home range and the core area (square-root-transformed; for all n = 32 observations from 10 groups and 9 years), we used a LMM [86] per response variable with either group size or number of males as the test predictor. To test whether core area exclusivity was higher than home range exclusivity, we used a LMM [86] (n = 64 observations from 9 groups, as much as 18 years and 32 combinations of group and year). The response variable was the percent of the exclusively used annual home range and core area and as test predictor we included whether the estimate was from the core area or not as a factor with 2 levels (yes = core area and no = home range; see also Additional file 4).

#### Statistical analyses

We fitted all models in R 3.4.3 [77] with Gaussian error structure and identity link [86] and implemented them using the functions lmer of the lme4 package [90]. All predictor variables were z-transformed to a mean of zero and a standard deviation of one [91]. To control for repeated observations, we included group ID and year ID as random effects in all models. Additionally, for the models testing whether core area fidelity and exclusivity were higher than home range fidelity and exclusivity, we also included the combination of group and year as a random effect. To keep error I rate at the nominal level of 5%, we included random slopes in all models where applicable (Additional file 5) [92, 93]. To check for the assumption of normally distributed and homogenous residuals, we visually inspected qqplots and the residuals plotted against fitted values. We found no violations. We investigated model stability by excluding each level of the random effects one at a time, including one group that foraged on crops outside the park. Comparing estimates for each predictor with those obtained for the full data set suggested no influential levels of random effects (Additional file 3). To investigate collinearity issues of the models having more than one test predictor, we determined Variance Inflation Factors (VIF) [94] using the function vif of the package car [95]. We applied the function to a model lacking the random effects and found no issues (maximum VIF across all models = 1.16). To establish significance of the full model compared to the null model lacking the test predictors (*p* < 0.05), we used a likelihood ratio test [96]. For the models having more than one test predictor, we determined individual *p* values based on likelihood ratio tests, which compared the full model with respective reduced models excluding the test predictors one at a time [93].

## Supplementary information

**Additional file 1.** Annual kernel home range and core area sizes for the Bwindi gorilla study groups.

**Additional file 2.** Summary of the mixed model results investigating the impact of group size and location as a proxy for local food availability on annual home range and core area size in Bwindi mountain gorillas.

**Additional file 3.** Tables of model stability of all investigated models. Discussion of the inclusion of Nkuringo Group, which ranged outside of the national park for a notable amount of the study.

**Additional file 4.** Similarity in space use between two neighboring groups using Bhattacharyya affinity of any two neighboring groups in the same year.

**Additional file 5.** Random slope structures of all investigated models.

## Data Availability

The datasets used and analyzed during the current study are available from the corresponding author on reasonable request.

## Abbreviations

BA: Bhattacharyya affinity
GPS: Global positioning system
LMM: Linear mixed model
