# Supplementary material for: Using long-term ranging patterns to assess within-group and between-group competition in wild mountain gorillas
Source: BMC Ecol. 2020 Jul 16;20:40. doi: 10.1186/s12898-020-00306-6 (PMC7367404; doi:10.1186/s12898-020-00306-6)
Supplement: Supplementary file 1 — Additional file 1. Annual kernel home range and core area sizes for the Bwindi gorilla study groups. [file 12898_2020_306_MOESM1_ESM.docx]

**Additional file 1**

Table S7 Annual kernel home range and core area sizes for the Bwindi gorilla study groups.

| Group | Year | Annual home range size (km^2^) | Annual core area size (km^2^) |
| --- | --- | --- | --- |
| Bi | 2007 | 9.15 | 3.04 |
| Bi | 2008 | 10.39 | 3.60 |
| Bi | 2009 | 9.47 | 2.61 |
| Bi | 2010 | 10.14 | 3.12 |
| Bi | 2012 | 13.75 | 5.17 |
| Bi | 2016 | 7.73 | 2.74 |
| Bi | 2017 | 10.00 | 3.72 |
| Bu | 2012 | 5.35 | 1.69 |
| Ha | 2001 | 12.11 | 3.70 |
| Ha | 2002 | 15.07 | 4.70 |
| Ha | 2004 | 12.34 | 3.71 |
| Ha | 2005 | 15.91 | 4.93 |
| Ha | 2006 | 16.37 | 4.42 |
| Ha | 2007 | 12.52 | 3.24 |
| Ha | 2008 | 14.30 | 4.41 |
| Ha | 2009 | 13.30 | 3.57 |
| Ha | 2010 | 13.82 | 3.51 |
| Ha | 2011 | 12.05 | 3.56 |
| Ha | 2012 | 14.85 | 4.48 |
| Kah | 2011 | 8.53 | 2.64 |
| Kah | 2012 | 9.38 | 3.06 |
| Ky | 1999 | 15.73 | 4.65 |
| Ky | 2000 | 15.25 | 4.71 |
| Ky | 2001 | 22.91 | 7.23 |
| Ky | 2002 | 16.52 | 5.63 |
| Ky | 2003 | 14.24 | 4.47 |
| Ky | 2004 | 17.13 | 5.93 |
| Ky | 2005 | 13.10 | 3.90 |
| Ky | 2006 | 10.80 | 3.85 |
| Ky | 2007 | 11.51 | 3.80 |
| Ky | 2008 | 10.71 | 3.29 |
| Ky | 2009 | 11.48 | 3.87 |
| Ky | 2010 | 11.06 | 2.85 |
| Ky | 2011 | 9.22 | 2.82 |
| Ky | 2012 | 14.37 | 5.23 |
| Ky | 2013 | 14.69 | 4.49 |
| Ky | 2014 | 9.24 | 3.12 |
| Ky | 2015 | 11.37 | 3.75 |
| Ky | 2016 | 9.27 | 3.05 |
| Ky | 2017 | 6.46 | 1.91 |
| Mi | 2012 | 4.80 | 1.79 |
| Mk | 2016 | 7.23 | 2.34 |
| Mk | 2017 | 8.73 | 3.19 |
| Mu | 2002 | 6.87 | 1.56 |
| Mu | 2004 | 4.11 | 1.40 |
| Mu | 2005 | 4.23 | 1.48 |
| Mu | 2007 | 4.64 | 1.23 |
| Mu | 2008 | 5.26 | 1.39 |
| Mu | 2009 | 4.80 | 1.42 |
| Mu | 2012 | 5.55 | 1.61 |
| Nk | 2004 | 8.94 | 3.10 |
| Nk | 2005 | 11.46 | 3.94 |
| Nk | 2006 | 12.57 | 4.21 |
| Nk | 2007 | 9.50 | 2.93 |
| Nk | 2008 | 9.14 | 2.95 |
| Nk | 2009 | 9.14 | 2.40 |
| Nk | 2012 | 9.75 | 3.18 |
| Ns | 2012 | 11.34 | 3.96 |
| Or | 2010 | 11.47 | 2.99 |
| Or | 2011 | 7.21 | 1.61 |
| Or | 2012 | 8.16 | 2.67 |
| Or | 2014 | 7.35 | 2.17 |
| Or | 2015 | 13.13 | 4.11 |
| Or | 2016 | 6.89 | 2.22 |
| Or | 2017 | 9.39 | 3.26 |
| Ru | 2004 | 7.57 | 1.77 |
| Ru | 2005 | 7.05 | 1.89 |
| Ru | 2006 | 9.17 | 2.27 |
| Ru | 2007 | 4.96 | 1.06 |
| Ru | 2012 | 7.58 | 1.93 |
