# Supplementary material for: Using long-term ranging patterns to assess within-group and between-group competition in wild mountain gorillas
Source: BMC Ecol. 2020 Jul 16;20:40. doi: 10.1186/s12898-020-00306-6 (PMC7367404; doi:10.1186/s12898-020-00306-6)
Supplement: Supplementary file 2 — Additional file 2. Summary of the mixed model results investigating the impact of group size and location as a proxy for local food availability on annual home range and core area size in Bwindi mountain gorillas. [file 12898_2020_306_MOESM2_ESM.docx]

**Additional file 2**

Table S8 Summary of the mixed model results investigating the impact of group size and location as a proxy for local food availability on annual home range and core area size in Bwindi mountain gorillas.

| Response variable | Annual home range size | | | | Annual core area size | | | |
| --- | --- | --- | --- | --- | --- | --- | --- | --- |
| Predictor variable | Est | SE | χ^2^ | P | Est | SE | χ^2^ | P |
| Intercept | 2.982 | 0.179 | ^a^ | ^a^ | 1.602 | 0.116 | ^a^ | ^a^ |
| Group size^b^ | 0.160 | 0.087 | 2.57 | 0.109 | 0.092 | 0.042 | 3.61 | 0.057 |
| Group size squared | -0.082 | 0.053 | 1.85 | 0.174 | -0.040 | 0.026 | 2.08 | 0.150 |
| Location(Ruhija)^c,d^ | 0.377 | 0.212 | ^a^ | ^a^ | 0.276 | 0.143 | ^a^ | ^a^ |
| Location(Rushaga)^c,d^ | 0.127 | 0.268 | ^a^ | ^a^ | 0.132 | 0.166 | ^a^ | ^a^ |

For each predictor variable, the estimates (Est), standard error (SE), the chi-square value χ^2^ (df=2), and the p‑values are shown. Significant results (p < 0.05) are indicated in bold.

^a^Not shown because of having a very limited interpretation.

^b^Group size was z-transformed to a mean of zero and standard deviation (sd) of one; original mean (sd) was: 12.59 (3.33). The transformed variable was squared.

^c^Proxy for food availability.

^d^Location was dummy coded with Buhoma being the reference category.
