# Supplementary material for: Using long-term ranging patterns to assess within-group and between-group competition in wild mountain gorillas
Source: BMC Ecol. 2020 Jul 16;20:40. doi: 10.1186/s12898-020-00306-6 (PMC7367404; doi:10.1186/s12898-020-00306-6)
Supplement: Supplementary file 3 — Additional file 3. Tables of model stability of all investigated models. Discussion of the inclusion of Nkuringo Group, which ranged outside of the national park for a notable amount of the study. [file 12898_2020_306_MOESM3_ESM.docx]

**Additional file 3**

Model stability of all investigated models

**Table S1** Model stability of the mixed model results investigating the impact of group size on annual home range and core area size in Bwindi mountain gorillas. For each model, we show the estimates (Est) from the full model and the minimum (Min) and the maximum (Max) of the estimates obtained when excluding levels of random effects one at a time.

| Response variable | Annual home range size | | | Annual core area size | | |
| --- | --- | --- | --- | --- | --- | --- |
| Predictor variable | Est | Min | Max | Est | Min | Max |
| Intercept | 3.183 | 3.007 | 3.281 | 1.760 | 1.649 | 1.820 |
| Group size | 0.158 | 0.056 | 0.208 | 0.084 | 0.044 | 0.128 |
| Group size squared | -0.090 | -0.178 | -0.029 | -0.051 | -0.075 | -0.016 |

Group size was z-transformed to a mean of zero and standard deviation (sd) of one; original mean (sd) was: 12.59 (3.33)

**Table S2** Model stability of the mixed model results investigating the impact of group size on annual home range and core area fidelity in Bwindi mountain gorillas. For each model, we show the estimates (Est) from the full model and the minimum (Min) and the maximum (Max) of the estimates obtained when excluding levels of random effects one at a time.

| Response variable | Annual home range fidelity (BA) | | | Annual core area fidelity (BA) | | |
| --- | --- | --- | --- | --- | --- | --- |
| Predictor variable | Est | Min | Max | Est | Min | Max |
| Intercept | 0.581 | 0.557 | 0.603 | 0.215 | 0.204 | 0.226 |
| Within-groups effect of group size | -0.002 | -0.021 | 0.018 | -0.005 | -0.023 | 0.000 |
| Between-groups effect of group size | -0.031 | -0.046 | -0.019 | -0.036 | -0.041 | -0.025 |

BA= Bhattacharyya affinity

Group size was z-transformed to a mean of zero and standard deviation (sd) of one; original mean (sd) was: 13.29 (2.75)

**Table S3** Model stability of the mixed model results investigating whether core area fidelity was higher than home range fidelity in Bwindi mountain gorillas. The table shows the estimates (Est) from the full model and the minimum (Min) and the maximum (Max) of the estimates obtained when excluding levels of random effects one at a time.

| Response variable | Annual home range and core area fidelity (BA) | | |
| --- | --- | --- | --- |
| Predictor variable | Est | Min | Max |
| Intercept | 0.595 | 0.575 | 0.617 |
| Core area (yes/no) | -0.372 | -0.384 | -0.361 |

BA= Bhattacharyya affinity

**Table S4** Model stability of the mixed model results investigating the impact of group size on the size of the exclusively used part of both the annual home range and core area in Bwindi mountain gorillas. For each model, we show the estimates (Est) from the full model and the minimum (Min) and the maximum (Max) of the estimates obtained when excluding levels of random effects one at a time.

| Response variable | Annual home range exclusivity | | | Annual core area exclusivity | | |
| --- | --- | --- | --- | --- | --- | --- |
| Predictor variable | Est | Min | Max | Est | Min | Max |
| Intercept | 20.312 | 18.687 | 22.902 | 13.961 | 13.518 | 14.832 |
| Group size | 4.210 | 2.825 | 7.797 | 1.823 | 1.318 | 3.084 |

Group size was z-transformed to a mean of zero and standard deviation (sd) of one; original mean (sd) was: 11.10 (3.39)

**Table S5** Model stability of the mixed model results investigating the impact of number of males per group on the size of the exclusively used part of both the annual home range and core area in Bwindi mountain gorillas. For each model, we show the estimates (Est) from the full model and the minimum (Min) and the maximum (Max) of the estimates obtained when excluding levels of random effects one at a time.

| Response variable | Annual home range exclusivity | | | Annual core area exclusivity | | |
| --- | --- | --- | --- | --- | --- | --- |
| Predictor variable | Est | Min | Max | Est | Min | Max |
| Intercept | 19.573 | 18.064 | 23.207 | 14.433 | 13.425 | 16.687 |
| Number of males | 1.445 | 0.788 | 8.288 | 1.928 | 1.210 | 3.999 |

Number of males was z-transformed to a mean of zero and standard deviation (sd) of one; original mean (sd) was: 2.57 (1.54)

**Table S6** Model stability of the mixed model results investigating whether core area exclusivity was higher than home range exclusivity in Bwindi mountain gorillas. The model shows the estimates (Est) from the full model and the minimum (Min) and the maximum (Max) of the estimates obtained when excluding levels of random effects one at a time.

| Response variable | Percent annual home range and core area exclusivity | | |
| --- | --- | --- | --- |
| Predictor variable | Est | Min | Max |
| Intercept | 47.973 | 43.394 | 54.639 |
| Core area (yes/no) | 28.083 | 25.439 | 30.543 |

Ranging outside the National Park

One of the twelve groups in our study had an atypical home range shape that included foraging in plantations and uncultivated land outside of the park (see the Nkuringo group in Figure 1; Seiler and Robbins, 2016). The availability of crops may alter the intensity of between-group and within-group competition compared to groups that range primarily in the forest, due to potential differences in the distribution and abundance of food resources. The renewal rates for crops could also differ from foods within the park, which might affect home range fidelity. Therefore, to examine whether the Nkuringo group had an unusual influence on our results, we performed post hoc evaluations of our model stability tests that had removed one group at a time from each model. To characterize the impact of each group on the parameter estimates in the models (and their 95% confidence intervals), we calculated the absolute relative difference between each value from the full dataset, versus the corresponding value when a group was removed. In the model for home range size, for example, the coefficient for group size was 0.158 with the full dataset, and it equaled 0.163 when the Bitukura group was removed, so the impact of removing the Bitukura group was abs((0.158-0.163)/0.163) = 0.0091. For the parameter estimates in the models, the impact of excluding Nkuringo was below the average impact of excluding other groups, so it did not seem to have an unusual influence on those results. For the 95% confidence intervals of those parameter estimates, the impact of excluding Nkuringo was above the average impact of excluding other groups, but it was still less than two other groups in the study. We also examined the changes in p‑values when each group was removed from the models. Two p‑values were no longer statistically significant when we removed the Nkuringo group from the models. The overall p‑value for core area size increased from 0.040 to 0.055, and p‑value for core area fidelity increased from 0.047 to 0.088. However, those p‑values also exceeded 0.05 when we removed three other groups from the model for core area size (one at a time), and when we removed five other groups from the model for core area fidelity. Thus, the loss of statistical significance does not necessarily reflect an influence of crop raiding. Instead, the changes may partially illustrate how reductions in sample sizes can diminish the statistical power of models that were only marginally significant to begin with. It is also not surprising that some data points would strengthen a correlation while others weaken it. Removing the Nkuringo group did not cause any p‑values to become significant, so we found no evidence that crop raiding could be obscuring a relationship in our data. Finally, the Nkuringo group does not appear to be an outlier or have excessive leverage in Figures 3 & 4. Overall, these post hoc evaluations provided no evidence to support the assertion that the Nkuringo group is a major problem in our study. It may be important to note, however, that an invalid hypothesis does not suddenly become valid because the data do not disprove it.
