# Supplementary material for: Using long-term ranging patterns to assess within-group and between-group competition in wild mountain gorillas
Source: BMC Ecol. 2020 Jul 16;20:40. doi: 10.1186/s12898-020-00306-6 (PMC7367404; doi:10.1186/s12898-020-00306-6)
Supplement: Supplementary file 4 — Additional file 4. Similarity in space use between two neighboring groups using Bhattacharyya affinity of any two neighboring groups in the same year. [file 12898_2020_306_MOESM4_ESM.docx]

**Additional file 4**

Similarity in space use between two neighboring groups using Bhattacharyya affinity of any two neighboring groups in the same year.

In addition to estimating the size and the percentage of the annual home range and core area that was not shared with any neighboring group, we compared the similarity of utilization distribution of any two neighboring groups to capture variation of space use within the home range between two neighbors. Using the package adehabitatHR [1] in R 3.4.3 [2], we determined the Bhattacharyya affinity (BA) [3] of any two neighboring groups in the same year (n = 54). A value of one indicates identical utilization distributions between two neighboring groups while zero indicates no similarity.

To test whether similarity in space use of the annual home ranges of two neighboring groups was higher than similarity in space use of the core area, we used a LMM [4]. The response variable was the BA of the annual home range for any two neighboring groups and the BA of the annual core areas of the same group dyads (square-root-transformed; n = 54 observations from 10 group dyads, as much as 9 years and 27 combinations of dyad and year). As test predictor we included whether the estimate was from the core area or not as a factor with two levels (yes = core area and no = home range). We included the random slopes of the dummy coded and z-transformed factor for core area (yes = core area and no = home range) within group dyad ID, year ID and a combination of group dyad and year ID. For details on the statistical analyses, please see main document (Statistical analyses).

We found that the mean BA of the annual home ranges between neighboring groups was 0.26 ± SE 0.04 (range 0.02-0.61) and the mean BA of the annual core areas between neighboring groups was 0.05 ± 0.01 (range 0-0.6). Lastly, the BA of the annual home ranges between any two neighboring groups was significantly lower than of the annual core areas between the same group dyads (Est ± SE = -0.307 ± 0.023, χ^2^ = 29.962, df = 1, p < 0.001).

These results suggest that core area use was significantly less similar between neighboring groups than home range use. However, the low BA values indicate that the similarity in home range space use between any two neighboring groups was low (mean BA: 0.26) and very low to absent for the annual core areas (mean BA: 0.05). This supports our finding that annual core areas were more exclusive than the respective home ranges.

References

1. Calenge C. The package “adehabitat” for the R software: A tool for the analysis of space and habitat use by animals. Ecological Modelling. 2006;197:516–9.

2. R Core Team. R: A language and environment for statistical computing. Vienna, Austria: R Foundation for Statistical Computing; 2017. https://www.R-project.org.

3. Bhattacharyya A. On a measure of divergence between two statistical populations defined by their probability distributions. Bulletin of the Calcutta Mathematical Society. 1943;35:99–109.

4. Baayen RH. Analyzing Linguistic Data: A Practical Introduction to Statistics using R. Cambridge: Cambridge University Press; 2008.
