# Supplementary material for: Using long-term ranging patterns to assess within-group and between-group competition in wild mountain gorillas
Source: BMC Ecol. 2020 Jul 16;20:40. doi: 10.1186/s12898-020-00306-6 (PMC7367404; doi:10.1186/s12898-020-00306-6)
Supplement: Supplementary file 5 — Additional file 5. Random slope structures of all investigated models. [file 12898_2020_306_MOESM5_ESM.docx]

**Additional file 5**

Random slope structures of all investigated models

For both the annual home range and the core area size model, we included the random slopes of group size and group size squared within group ID and year ID. The models testing annual home range and core area fidelity (BA) contained the random slopes of the within-groups effect of group size within group ID and both the within-groups effect and the between-groups effect of group size within both years ID. For the model investigating core area versus home range fidelity, we included the random slopes of the dummy coded and z-transformed (to a mean of zero and a standard deviation of one) factor for core area (yes = core area and no = home range) within group ID, both years ID and a combination of group and both years ID. The models testing the sizes of the exclusively used part of the home range and the core area contained the random slopes of the within-groups effect of group size and number of males, respectively, within group ID and year ID. For the model examining the percent of exclusively used annual home range versus core area, we included the random slopes of the dummy coded and z-transformed factor for core area (yes = core area and no = home range) within group ID, year ID and a combination of group and year ID.
